# A Scalable, Easy-to-Deploy Protocol for Cas13-Based Detection of SARS-CoV-2 Genetic Material

**DOI:** 10.1128/JCM.02402-20

**Published:** 2021-03-19

**Authors:** Jennifer N. Rauch, Eric Valois, Sabrina C. Solley, Friederike Braig, Ryan S. Lach, Morgane Audouard, Jose Carlos Ponce-Rojas, Michael S. Costello, Naomi J. Baxter, Kenneth S. Kosik, Carolina Arias, Diego Acosta-Alvear, Maxwell Z. Wilson

**Affiliations:** a University of California, Santa Barbara, Department of Molecular, Cellular, and Developmental Biology, Santa Barbara, California, USA; b Neuroscience Research Institute, University of California, Santa Barbara, Santa Barbara, California, USA; c Center for BioEngineering, University of California, Santa Barbara, Santa Barbara, California, USA; St. Jude Children's Research Hospital

**Keywords:** CRISPR, Cas13, COVID-19, point of care, SARS-CoV-2, testing

## Abstract

The COVID-19 pandemic has created massive demand for widespread, distributed tools for detecting SARS-CoV-2 genetic material. The hurdles to scalable testing include reagent and instrument accessibility, availability of highly trained personnel, and large upfront investment. Here, we showcase an orthogonal pipeline we call CREST (Cas13-based, rugged, equitable, scalable testing) that addresses some of these hurdles. Specifically, CREST pairs commonplace and reliable biochemical methods (PCR) with low-cost instrumentation, without sacrificing detection sensitivity. By taking advantage of simple fluorescence visualizers, CREST allows a binary interpretation of results. CREST may provide a point-of-care solution to increase the distribution of COVID-19 surveillance.

## Abstract (precis)

The COVID-19 pandemic has created massive demand for widespread, distributed tools for detecting SARS-CoV-2 genetic material. The hurdles to scalable testing include reagent and instrument accessibility, availability of highly trained personnel, and large upfront investment.

## INTRODUCTION

The coronavirus 2019 (COVID-19) pandemic presents the world with an unprecedented public health challenge. The lack of COVID-19 symptoms in a significant proportion (estimates range from 18% to 29%) of individuals infected with severe acute respiratory syndrome coronavirus 2 (SARS-CoV-2) fuels covert transmission of the virus (1, 2). Even in cases in which symptoms do occur, the virus can be transmitted before symptom onset (3, 4). The high proportion of asymptomatic or mildly symptomatic and highly infectious individuals, combined with the risk of severe disease outcomes and mortality rates ranging from 1.38% to as high as 20% in people 80 years and older, places an unprecedented burden on our health care system (5, 6).

A range of mitigation efforts have been implemented across the globe to slow the transmission and “flatten the curve,” including sheltering in place and the requirement for face covering in public. However, without widely available and accurate testing, it remains unclear exactly how effective these methods are at curbing the spread of the virus. In addition, these risk mitigation efforts have a catastrophic economic and social impact, which makes long-term compliance challenging until a vaccine or treatment is widely available (7). To reduce the socioeconomic burden, we need to implement effective containment measures, such as contact tracing, quarantine of confirmed cases, and enhanced surveillance, all of which depend on data obtained from widespread testing (8). Proactive, prevalent, and regular testing have been effective measures for the control of COVID-19 in countries such as South Korea and Taiwan and will be required for the establishment of effective strategies for the relaxation of social-distancing measures worldwide (9, 10).

A critical hurdle to deploying massively widespread, recurrent testing is the availability of reagents and specialized equipment. Suggested solutions to make sample collection scalable include laboratory-made viral transport media and 3D-printed swabs and bypassing biochemical RNA extraction through heat and chemical extraction techniques (11–14). However, there have been few end-to-end solutions to SARS-CoV-2 testing that fulfill the requirements of being immediately scalable and low in cost without sacrificing sensitivity. Here, we focused on developing a CRISPR (clustered regularly interspaced palindromic repeats)-based SARS-CoV-2 RNA detection method that is low in cost, highly sensitive, and easy to deploy at sites with minimal infrastructure.

Cas12 and Cas13 (CRISPR-based) methods have been transformative with regard to pathogen detection. They are sensitive and, when coupled to isothermal amplification methods and lateral flow immunochromatography detection, have been made field deployable. These methods are promising tools for the detection of SARS-CoV-2 (15–18). However, because of the global demand for testing, key reagents in these protocols are difficult to obtain. To lower the barrier to COVID-19 diagnostics, we devised a method we call CREST (Cas13-based, rugged, equitable, scalable testing). CREST addresses three of the main hurdles—reagent accessibility, equipment availability, and cost—that limit the scalability of Cas13-based testing, by taking advantage of widely available enzymes, low-cost thermocyclers, and easy-to-use fluorescent visualizers. Moreover, CREST is equivalent in sensitivity to the gold standard reverse transcription quantitative PCR (RT-qPCR) method most often deployed for COVID-19 testing. With these advantages, CREST has the potential to facilitate early detection of positive cases, regular monitoring of individuals at high risk, and implementation of informed containment measures for infected individuals.

## METHODS AND RESULTS

To design a sensitive, low-cost, and easy-to-use SARS-CoV-2 detection method, we first identified critical steps that require limiting reagents, specific equipment, and highly trained individuals to perform them and thus present a barrier to testing at sites with limited infrastructure and resources. For CRISPR-Cas13-based methods, these steps include (i) the amplification of the target material prior to detection and (ii) the visualization of Cas13 activity by either colorimetric (immunochromatography) or fluorescent methods.

**FIG 1:**
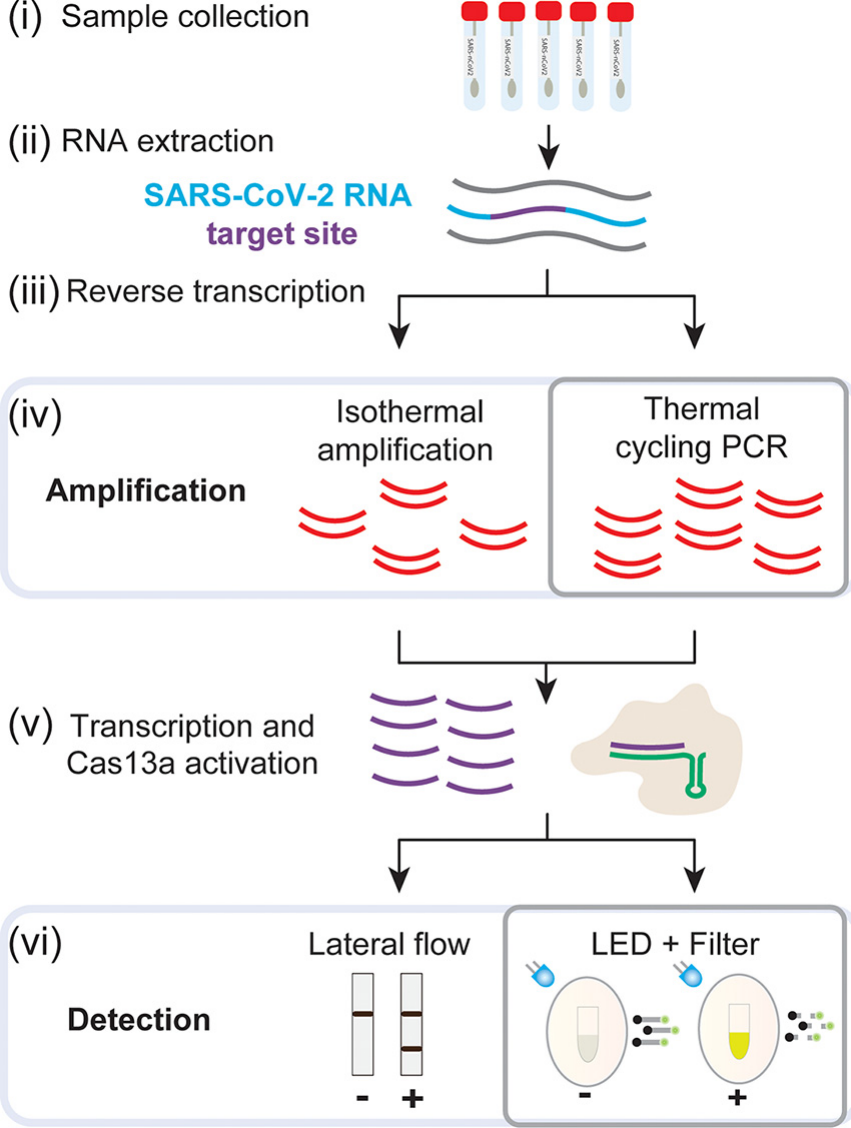
Overview of Cas13-based detection methods and CREST modifications. (i to iii) Standard sample collection, RNA extraction, and reverse transcription. (iv) Amplification using cost-effective *Taq* polymerase and portable thermocyclers instead of isothermal reactions. (v) Transcription and Cas13 activation are followed by fluorescence detection of dequenched poly(U) cleavage reporter visualized with a blue LED (∼495 nm) and orange filter or other fluorescence detection system.

**FIG 2:**
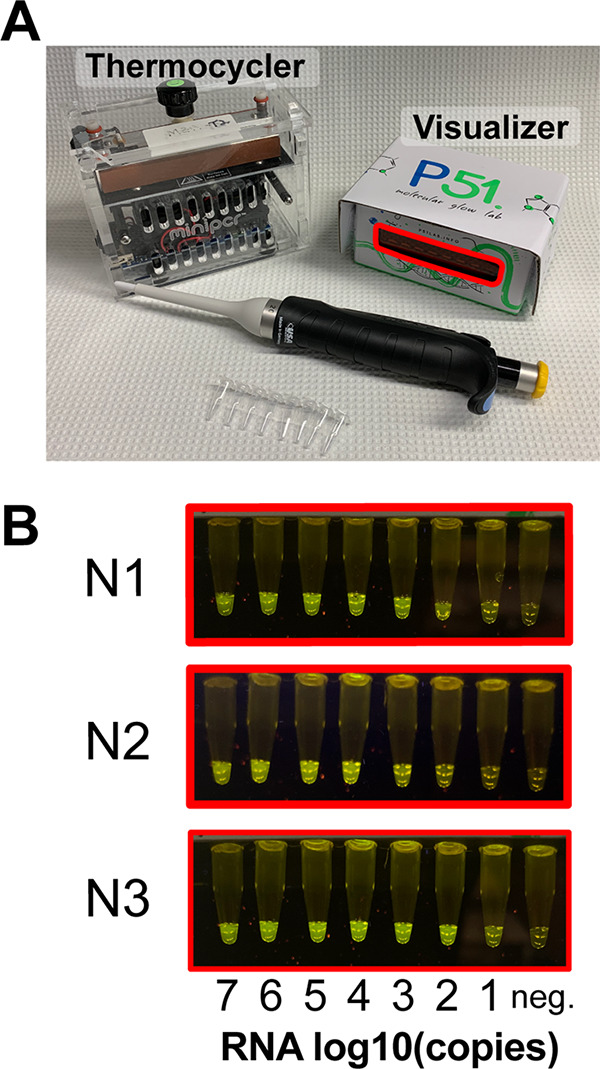
Detection of SARS-CoV-2 RNA using CREST. (A) The mini-PCR mini16 thermocycler and P51 molecular fluorescence visualizer used in this study. Both are portable, can be operated with batteries, and have minimal footprint. (B) Fluorescence visualization of N1, N2, and N3 synthetic targets using the P51 visualizer.

To lower the first barrier, we analyzed options for detection of specific SARS-CoV-2 genomic sequences upon enzymatic amplification (Fig. 1). The gold standard method relies on RT-qPCR (19). Quantitative detection is accomplished using specialized instruments that detect fluorescent probes which report the extent of amplification of the target sequence in real time. While sensitivity is high (on the order of tens of target molecules per microliter), the main limitation is the requirement of real-time thermocyclers, analysis software, and trained personnel for data interpretation. Limitations aside, the core of the technology—amplification of a target nucleic acid sequence by PCR—is robust and sensitive and makes use of a widely available enzyme, *Taq* polymerase. These advantages motivated us to pair PCR with CRISPR-based detection of viral sequences, an approach that has been successful for the detection of DNA sequences using Cas9 (20). The thermocyclers required for PCR are expensive, specialized instruments that are generally limited to professional laboratories. However, the recent “do-it-yourself biology” (DIYbio) movement has made PCR accessible through the creation of affordable, Bluetooth-enabled, field-ready thermocyclers, which can even be battery operated. These versatile thermocyclers can be used in unconventional environments and perform as well as traditional thermocyclers in moderate temperatures (21–23). We reasoned that these devices, such as the mini-PCR mini16, offer a low-cost solution for the amplification of the viral target material and can make COVID-19 testing widely available (Fig. 2).

**FIG 3:**
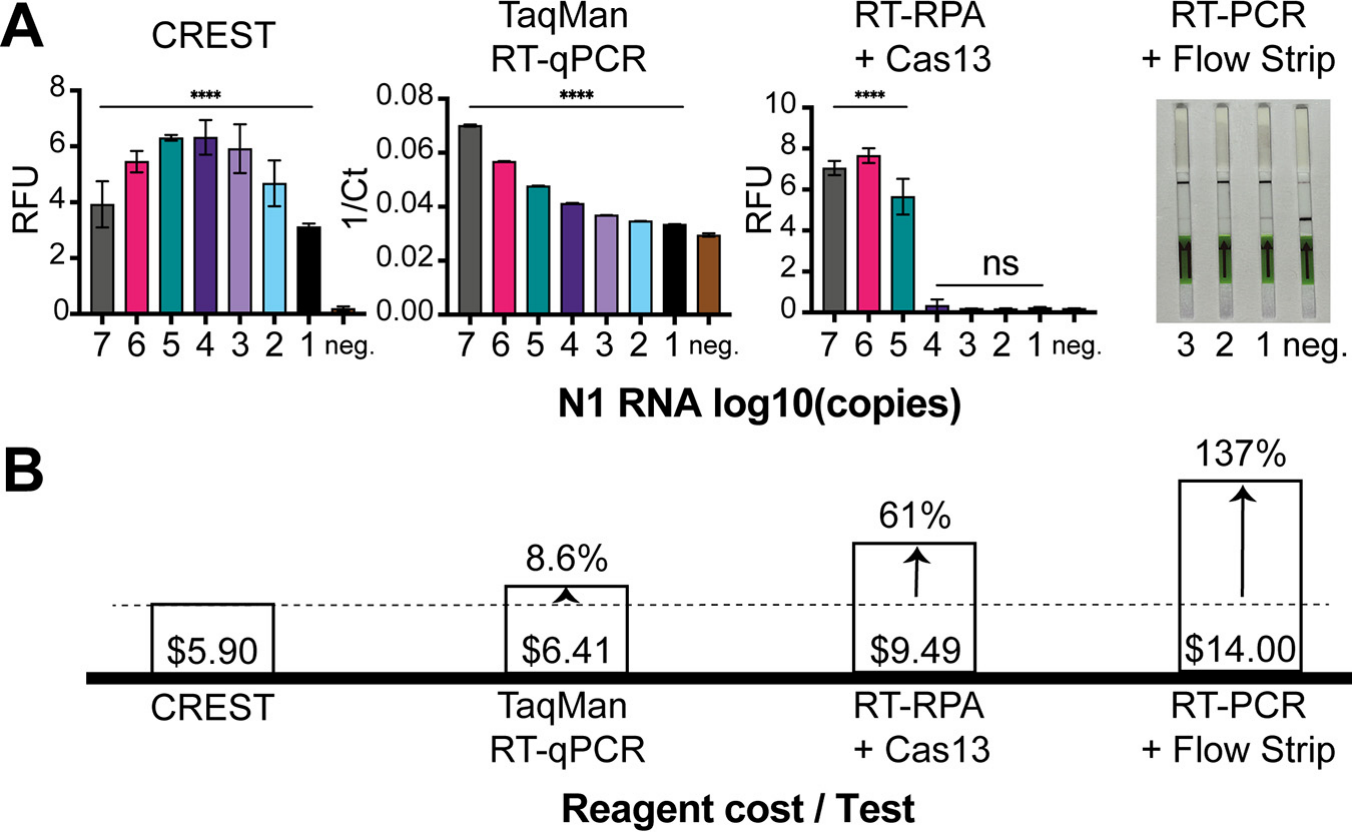
Comparative analysis of method sensitivity and reagent cost per test. (A) (Left) Comparison of method sensitivity using a quantitative fluorescence detection instrument. (Right) RT-PCR + Cas13 detection visualized with lateral flow strips. (B) Associated costs of reagents per test of each testing method (excluding up-front instrumentation costs). A test is defined as a single sample run in triplicate.

To reduce the second hurdle, the visualization of test outcomes, we explored options available for the detection of Cas13 activity. When bound to its target, Cas13 catalyzes the nonspecific cleavage of RNAs. This target-specific recognition can be detected in many ways, for example, either by lateral flow immunochromatography or by fluorescence visualization through the use of a fluorescein- and quencher-conjugated poly(U) RNA cleavage reporter. Lateral-flow test strips are a promising detection method. These strips utilize capillary action to move analytes through a solid support material striped with antibodies that can detect them, resulting in binary readouts. While they are simple to use and read—they may eventually enable in-home testing—their current availability is limited, and they are expensive relative to the cost per test (Fig. 3B; see also Data Set S1 in the supplemental material). For this reason, we sought an affordable, scalable, easy-to-interpret, solution for visualization of positive results. In the CREST protocol, we use a P51 cardboard fluorescence visualizer, powered by a 9-V battery, for the detection of Cas13 activity instead of immunochromatography (Fig. 2).

To validate a streamlined workflow with these devices, we measured the presence of the three viral sequences that correspond to the CDC RT-qPCR test (19). Briefly, we PAGE purified annealed synthetic DNA oligonucleotides flanked by an upstream T7 RNA polymerase promoter that encode sequences corresponding to the N1, N2, and N3 sites in the SARS-CoV-2 nucleocapsid gene (Table S3). Next, we transcribed the DNA *in vitro* to obtain target RNAs. After Cas13 purification and extensive optimization of the reaction conditions (Fig. S1 and S2), we used these targets to determine the detection limit of the CREST protocol and found that we could detect as few as 10 copies of a target RNA molecule per μl (Fig. 2B). This result shows that CREST has sensitivity comparable to that of the corresponding RT-qPCR in our hands, demonstrating the power of CREST for pairing a thermal cycling amplification step (PCR) with a linear amplification step (transcription), combined with enzymatic signal amplification through fluorescence detection. In addition, we calculated CREST’s limit of detection in negative human nasopharyngeal (NP) swabs with spiked heat-inactivated SARS-CoV-2 virus (ATCC VR-1986HK) to be 200 copies per μl (Fig. S3).

Next, we quantitively compared CREST’s sensitivity and cost to those of established methods. First, we compared it to RT-qPCR (one-step TaqMan assay) and found that, while being similarly sensitive, CREST’s reagents cost less than RT-qPCR’s even at the low scale of our pilot experiment (Fig. 3; Data Set S1). In addition, the up-front cost of CREST instrumentation is 30 to 50 times lower. Second, we compared the RT-PCR amplification step of CREST to Cas13-based protocols that utilize RT-recombinase polymerase amplification (RT-RPA). We found thermal cycling amplification (20 cycles) to be substantially more efficient with comparable amplification reaction times (Fig. 3A; Fig. S4). Moreover, in stark contrast to the proprietary, high-cost, relatively small-batch production rates of reagents required for RPA, *Taq* polymerase, which has been in high-volume production for decades and is a workhorse of modern molecular biology, is readily accessible and stable at room temperature and lowers costs significantly (Fig. 3B). Last, we compared lateral-flow test strip visualization to CREST, and while we found them as sensitive as fluorescent detection methods (Fig. 3A), their high cost and difficulty to obtain can limit their distribution and scaled use in this pandemic.

**FIG 4:**
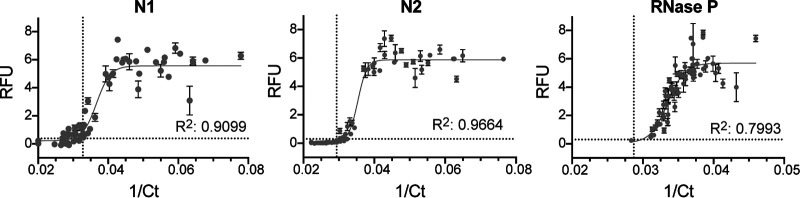
Comparative analysis between CREST and TaqMan for SARS-CoV-2 N1 and N2 sites and RNase P. De-identified human samples are shown as gray circles. Error bars show standard deviations (*n* = 4). Dotted lines indicate the detection threshold for each assay.

**FIG 5:**
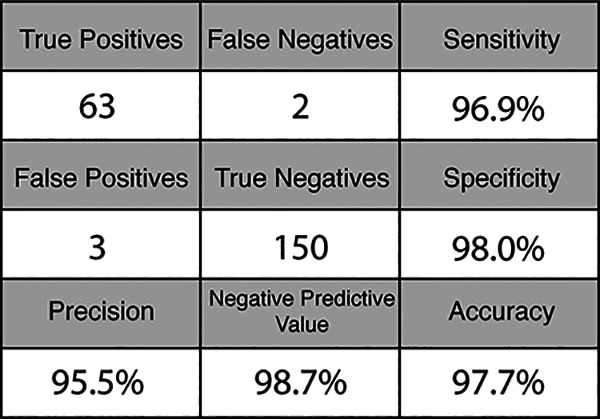
Confusion matrix of combined results from Santa Barbara County Department of Public Health, UCSF, and UCSB asymptomatic clinical sample sets.

To test the efficacy of CREST on human samples, we obtained 64 de-identified nasopharyngeal (NP) swabs from individuals collected through the Santa Barbara County Department of Public Health. We purified RNA from these samples, which were stored in viral transport medium, using a commercially available RNA extraction kit (QIAamp Mini Elute virus spin kit; Qiagen). We used this RNA as input for a parallel comparison between CREST and the CDC-recommended one-step TaqMan assay (our end-to-end CREST protocol is described in the supplemental material). Considering that CREST was designed to provide a binary outcome, we fit the CREST-to-TaqMan comparison to a sigmoid. We then calculated a goodness-of-fit *R* value between assays for detection of N1, N2, and RNase P (Fig. 4). These analyses revealed high concordance between CREST and TaqMan assays (*R*^2^ > 0.9 for SARS-CoV-2 genes and *R*^2^ > 0.79 for RNase P). Of note, CREST appears to be more sensitive than TaqMan for detection of N1, whereas the converse appears to be true for N2. We conducted two additional comparisons, one on 95 asymptomatic individuals at University of California, Santa Barbara (UCSB), where oropharyngeal (OP) self-sampling was carried out, and a second one on 30 positive and 30 negative NP/OP samples obtained from University of California, San Francisco (UCSF) (Fig. S6; Data Set S2). Taken together, the results show that CREST had a sensitivity of 97% and a specificity of 98% (Fig. 5).

Finally, while we designed CREST to be an accessible and scalable assay for detecting SARS-CoV-2, it still requires RNA extraction using commercial kits, which limits its widespread adoption. In a companion paper, we present a method called PEARL (precipitation-enhanced analyte retrieval) which uses common laboratory reagents to bypass this limitation (24). To lift the final obstacle to CREST’s accessibility, we coupled PEARL with CREST and found that commercial RNA extraction could be omitted (Fig. S7).

## DISCUSSION

The unparalleled spread of SARS-CoV-2 demands orthogonal solutions to expand testing capacities. Here, we describe an optimized Cas13-based protocol that uses accessible reagents and affordable equipment while maintaining sensitivity that is comparable to that of established testing methods. Considering the need for a rugged, equitable, and scalable test, we coupled spartan, Bluetooth-enabled thermocyclers that can be plugged into a battery and run using mobile device applications, with simple plastic-filter-based LED visualizers. Results can be simply documented with a smartphone camera and uploaded to the cloud, enabling distributed point-of-care (POC) testing. Operational costs could be further lowered by multiplexing the amplification of viral targets.

Current methods to detect SARS-CoV-2 presence or prior exposure in patient samples rely on detection of the viral genome or antibodies in nucleic acid and serological tests, respectively (24). The detection of viral genome sequences by RT-qPCR is recommended by public health organizations worldwide. However, these recommendations fail to account for the massive need for widespread testing. Implementing this method alone places a single large burden on the manufacturing capacity for RT-qPCR testing reagents and consumables, specialized instrumentation, and trained laboratory personnel, thus hampering widespread testing. Other POC diagnostic platforms still require specialized instrumentation and are low throughput (e.g., Abbott ID NOW).

These limitations underscore the need for alternative methods, such as CRISPR-based detection, to relieve some of the strain on the global supply chain for testing reagents. These state-of-the-art methods are scalable, do not entail specialized instrumentation, and require very little specialized training. CREST can be run, from RNA sample to result, with no need for AC power or a dedicated facility and with minimal handling, in ∼2 h. It also has a throughput comparable to that of RT-qPCR tests. Indeed, in a companion study, we processed ∼300 CREST samples per day with two operators and two thermocyclers (25).

We found CREST to have sensitivity comparable to that of RT-qPCR. Of the 218 clinical samples we tested with both of these methods, we identified 2 false-negative and 3 false-positive results by CREST. The 3 false-positive samples were reported as negative by RT-qPCR because the levels of the N1 target were below the detection threshold, although N2 detection by this method was positive. The 2 false-negative samples were above CREST’s detection threshold for N1 but not N2. Indeed, N1 CREST target detection was consistently slightly more sensitive than that for N2. In a clinical testing scenario, discordant results between N1 and N2 may be resolved by retesting. Finally, because CREST’s signal saturates even at low input levels (Fig. 2B), CREST offers the added advantage of binary result interpretation, similar to lateral-flow test strips, but at a fraction of the price, as it does not require the costly antibodies or antibody conjugates needed for lateral-flow immunochromatography.

By utilizing off-the-shelf components and an efficient purification protocol of Cas13, CREST’s costs remain close to those of RT-qPCR, one of the least expensive testing modalities currently available. We chose to compare CREST’s cost to that of Thermo Fisher’s general-purpose TaqPath reagent because of its validation by the CDC (19) and EUA status. However, purpose-built reaction mixes could make RT-qPCR costs closer to, or even lower than, that of CREST. Though Cas13 is not yet commercially available, we do not foresee its commercial-scale production to be a bottleneck. Indeed, we were able to purify enough protein from a 1-liter bacterial culture for more than 500,000 reactions, which leads us to conclude its cost per reaction is minimal. We expect Cas13 protein cost to be in the range of costs of other enzymes used in this study, between $0.05 and $0.50 per reaction. Other cost considerations include the labor involved in handling samples and setting up reactions.

CREST requires innovations before it can be fully POC capable. CREST entails two pipetting steps, one of which involves opening the reaction tube postamplification. This step increases the possibility of contaminating other samples (26). Removing these steps by either employing thermostable Cas variants that can survive the amplification reaction (27) or using disposable microfluidic cartridges with specialized reaction vessels to separate compartments (28) will be essential for establishing CREST’s full POC capability. Other requirements for field deployment of Cas13 include optimizing long-term enzyme storage conditions. Indeed, our Cas13 master mix is not affected significantly by undergoing numerous freeze-thaw cycles (Fig. S5), which increases CREST’s potential for field deployment by overcoming the need for professional laboratories.

Regular testing may be a key determinant impacting the ability to ascertain foci of infection and reduce false-negative rates. Recent studies document that in many individuals, even those with active symptoms, initial tests can be negative, while subsequent tests are positive, and intermittent detection of the virus in samples can also occur (29–32). Despite the biochemical reliability of currently available diagnostics, the initial quality of a sample and the viral load in the patient can affect the likelihood of a false result. Because of its low costs and ease of use, CREST could be employed for regular testing as well as for disambiguation of results obtained with established methods.

## Supplementary Material

Supplemental file 1

Supplemental file 2

Supplemental file 3

Supplemental file 4
