# Supplementary material for: A Scalable, Easy-to-Deploy Protocol for Cas13-Based Detection of SARS-CoV-2 Genetic Material
Source: J Clin Microbiol. 2021 Mar 19;59(4):e02402-20. doi: 10.1128/JCM.02402-20 (PMC8092748; doi:10.1128/JCM.02402-20)
Supplement: Supplemental file 2 [file JCM.02402-20-s0002.pdf]

CREST PROTOCOL

Follow biosafety guidelines for handling patient material as described here:
https://www.cdc.gov/coronavirus/2019-ncov/downloads/rt-pcr-panel-for-detection-instructions.pdf

A. RNA Extraction

We recommend the use of CDC approved kits for RNA extraction however we have found the QIAamp minElute virus spin kit (cat# 57704) or QIAamp Viral RNA Mini Kit (cat# 52904) performs satisfactorily in CREST. Bypassing RNA extraction has been proposed as a feasible alternative (1. https://science.sciencemag.org/content/360/6387/444.full, 2. https://www.biorxiv.org/content/10.1101/2020.04.22.056283v1.full.pdf)). In a companion paper (see reference 29 in the main text and here https://doi.org/10.1101/2020.06.29.178384) we developed simple method for bypassing RNA extraction.

B. Reverse Transcription (RT)

WARNING – CREST is sensitive and cross contamination is possible. We recommend having separate workstations for pre-amplification and post-amplification procedures.

1. Prepare the pre-amplification workstation by wiping surfaces and pipettes with RNase Away.
2. Calculate the amount of reactions (N) to assemble and multiply by 1.2 to account for 20% pipetting error. Thaw sufficient amounts of reagents for 1.2 X N reactions, listed in table below, on ice. Keep all reagents and reaction products on ice unless otherwise stated.
3. Prepare the RT master mix by adding reagents to a 0.2 or 0.5 mL tube in the order listed below:

**Reverse Transcription for N = 1**	**1X (μL)**
Gene Specific Primer Mix (5 μM each primer)	1.0
5X Reaction Buffer	2.0
dNTPs (10 mM each NTP)	1.0
RevertAid Reverse Transcriptase (200 U/μL)	0.5
Murine RNase inhibitor (40 U/ μL)	0.5
TOTAL VOLUME	5.0

4. Transfer 5 μL of master mix to a PCR tube on ice.
5. Add 5 μL of template RNA to PCR tube.
6. Mix gently by pipette (2 up-down strokes) and ensure that tube content is collected at the bottom of the tube.

7. Incubate at 42°C for 30 minutes in a miniPCR mini16 thermocycler, once complete immediately place the tubes on ice and proceed to PCR amplification.

B. PCR Amplification

1. Calculate the amount of reactions (N) to assemble and multiply by 1.2 to account for 20% pipetting error. Thaw sufficient amounts of reagents for 1.2 X N reactions, listed in table below, on ice. Keep all reagents and reaction products on ice unless otherwise stated.
2. Prepare PCR master mix by adding reagents to 0.2 mL PCR tubes in the order shown below:

**PCR Amplification for N = 1**	**<u>1X (µL)</u>**
Nuclease Free Water	17.0
5X Master Mix	5.0
Gene Specific Primer Mix (5 µM each primer)	1.0
TOTAL VOLUME	23.0

3. Carefully transfer 2µL from RT reaction in step A7 to tube containing PCR master mix.
4. Mix gently by pipette (2 up-down strokes) and ensure contents are collected at the bottom of the tube.
5. Place PCR tubes in a miniPCR mini16 thermocycler and amplify DNA using the following thermocycling program:

<table>
<tr><td>95°C</td><td>2 min</td><td></td></tr>
<tr><td>95°C</td><td>15 sec</td><td rowspan="3">20X</td></tr>
<tr><td>60°C</td><td>15 sec</td></tr>
<tr><td>72°C</td><td>15 sec</td></tr>
<tr><td>72°C</td><td>5 min</td><td></td></tr>
</table>

6. Upon completion of the PCR reaction place them on ice.
7. Take precautions to avoid amplicon cross-contamination. **Do not open the tubes containing the PCR products in pre-amplification work area.** Open the tubes in a designated post-amplification workstation.

C. Cas13 Detection

1. Calculate the amount of reactions (N) to assemble and multiply by 1.2 to account for 20% pipetting error. Thaw sufficient amounts of reagents for 1.2 X N reactions, listed in table below, on ice. Keep all reagents and reaction products on ice unless otherwise stated.
2. Dilute LwaCas13a to 63 μg/ml using Cas13a storage buffer (50 mM Tris pH 7.5, 600 mM NaCl, 5% Glycerol, 2 mM DTT).
3. Prepare a Cas13a detection master mix according to table below. We routinely assemble 5 μL reactions, as detailed in the table, in 0.2 mL PCR tube strips, but 20 μL reactions can also be used to increase visibility of results. Additionally, large batches of master mix can be pre-assembled and stored at -20°C. We have observed sustained activity of master mix upon three rounds of freeze/thaw.

**Cas13a Detection for N = 1**	**1x (μL)**
Ultra-pure H_2O	1.6
10X Cleavage Buffer (400 mM Tris pH 7.5, 10 mM DTT)	0.5
rNTPs (25 mM each)	0.2
Murine RNase Inhibitor (40 U/μL)	0.25
Cleavage Reporter (2 μM)	0.3
LwaCas13a (63 μg/ml)	0.5
T7 RNA Polymerase (50 U/μl)	0.15
Cas13 crRNA (2 μM)	0.05
$MgCl_2$ (100 mM)	0.45
TOTAL VOLUME	4.0

4. Carefully transfer 1 µL of the PCR reaction from step B7 to the tubes containing the Cas13a detection master mix. Ensure transfer of the correct PCR products to the corresponding tubes with the correct crRNA.
5. Mix gently by pipette (2 up-down strokes) and collect contents at the bottom of the tube.
6. Incubate tubes at 37°C for 30 minutes.
7. Visualize CREST reactions using P51 fluorescence visualizer*.

***NOTE:** Fluorescence can also be monitored quantitatively in a plate reader or in a qPCR instrument.

List of Reagents

1. RevertAid Reverse Transcriptase. Thermo Fisher Scientific, Cat # EP0442
2. Deoxynucleotide (dNTP) Solution Mix 10 mM each Nt. New England Biolabs Inc, Cat # N0447L
3. RNase Inhibitor, Murine. New England Biolabs Inc, Cat # M0314L
4. *Taq* 5X Master Mix. New England Biolabs Inc, Cat # M0285L
5. Ribonucleotide Solution Set, 50 µmol of each. New England Biolabs, Cat # N0450L
6. NxGen T7 RNA Polymerase. Lucigen, Cat # 30223-2

7. CRISPR-RNA (crRNA) and Cleavage Reporter:

gRNA_N1	GAU UUA GAC UAC CCC AAA AAC GAA GGG GAC UAA AAC AGG GUC CAC CAA ACG UAA UGC GGG GUG C	Integrated DNA Technology (IDT)
gRNA_N2	GAU UUA GAC UAC CCC AAA AAC GAA GGG GAC UAA AAC GCU GAA GCG CUG GGG GCA AAU UGU GCA A	Integrated DNA Technology (IDT)
gRNA_N3	GAU UUA GAC UAC CCC AAA AAC GAA GGG GAC UAA AAC UAG CAG GAU UGC GGG UGC CAA UGU GAU C	Integrated DNA Technology (IDT)
gRNA_RNA	GAU UUA GAC UAC CCC AAA AAC GAA GGG GAC UAA AAC GUC CGC GCA GAG CCU UCA GGU CAG AAC C	Integrated DNA Technology (IDT)
Cleavage Reporter	56-FAM-UU UUU UU-3IABkFQ	Integrated DNA Technology (IDT)
